# A Rare Case of Cardiac Recovery after Acute Myocarditis from West Nile Virus Infection: A Review of the Current Literature

**DOI:** 10.1155/2022/8517728

**Published:** 2022-09-28

**Authors:** KaChon Lei, Wilbur Ji, Bhavana Bhaya, Chowdhury Ahsan

**Affiliations:** ^1^Kirk Kerkorian School of Medicine at University of Nevada, Las Vegas, Department of Internal Medicine, USA; ^2^Kirk Kerkorian School of Medicine at University of Nevada, Las Vegas, Division of Cardiovascular Medicine, USA

## Abstract

West Nile Virus (WNV) myocarditis is nearly fatal, according to the current medical literature. We report a previously healthy 37-year-old Caucasian male who presented to our facility with two days of progressive lower extremity weakness, fever, edema, and shortness of breath found to have left ventricular global hypokinesis with an ejection fraction of less than 25%, consistent with acute viral myocarditis. He also has concomitant WNV meningoencephalitis due to his altered mentation. He was found to have a positive serum WNV IgM suggestive of a diagnosis of WNV myocarditis. He was intubated and was placed on vasoactive pressors for supportive care due to evidence of mixed cardiogenic and septic shock. After two weeks of hemodynamic support, we discovered a near-complete cardiac recovery, as shown on a repeat transthoracic echocardiography (TTE) and a normalized mean arterial blood pressure. This is a unique case report because near fatality is often associated with WNV myocarditis secondary to tachyarrhythmia, and there are currently no documented cases that are suggestive of cardiac recovery from the current literature.

## 1. Introduction

West Nile Virus, a mosquito-borne RNA flavivirus, is associated with neuroinvasive disease and systemic illness. Although 80% of infected individuals are asymptomatic, it is the reported etiology of viral encephalitis in the United States (approximately less than 1% of all cases) [1]. The prognosis of WNV infection is controversial, but only approximately 30% of patients will develop systemic symptoms, including fever, headache, ataxia, and weakness [2]. The mortality of WNV infection is 2%, according to the CDC. Young age appears to be the single most significant protective prognostic factor, in addition to immunocompetency. However, the progression of the disease to WNV myocarditis has demonstrated poor clinical outcomes due to rapidly deteriorating cardiorespiratory status and its associated conduction abnormalities [1–3]. Documented cases of WNV myocarditis are common in domesticated animals, with only a few cases reported in humans. WNV myocarditis is not associated with a specific arrhythmia; however, observational studies have suggested that 2nd and 3rd-degree heart blocks are prevalent in WNV infected hosts [4]. Once myocarditis is apparent in the clinical picture, tachyarrhythmias appear inevitable, and the mortality rate approaches 100%. A previous case report showed that patients with WNV myocarditis are at risk of developing idioventricular rhythm and asystole [3]. We herein report a rare case of a 37-year-old male with WNV myocarditis, who presented with shock physiology and had cardiac recovery after receiving supportive care, IVIG, and plasmapheresis.

## 2. Case Report

**Figure 1:**
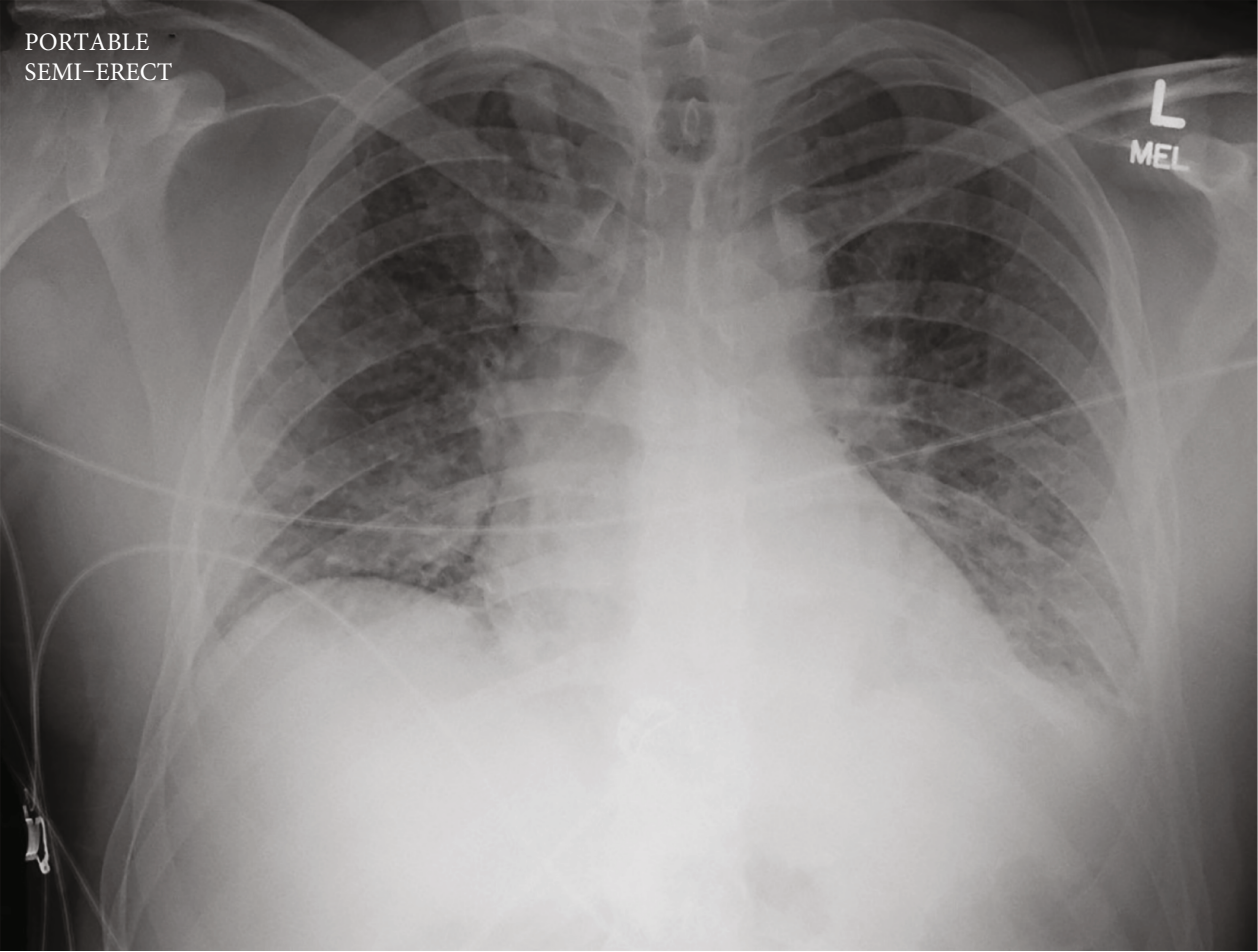
Chest X-ray on admission shows bilateral pulmonary infiltrates concerning for pulmonary edema vs. pneumonia.

**Figure 2:**
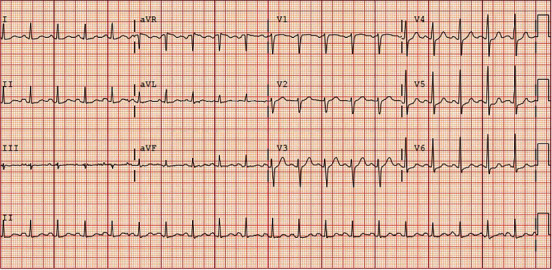
EKG on admission shows sinus tachycardia otherwise normal rhythm with no significant ST changes.

**Figure 3:**
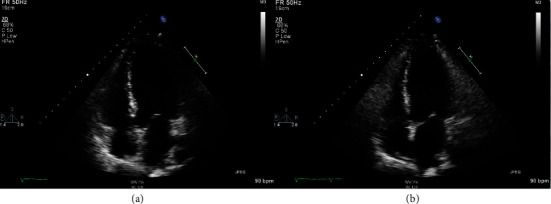
Four-chamber view of TTE demonstrates global wall motion hypokinesis and reduced LV systolic function on admission in the setting of a structurally normal heart on hospital day 3 after diuresis. (a) Four-chamber view during diastole. (b) Four-chamber view during systole with decreased contractility and minimal changes in LV size.

A 37-year-old Caucasian man with no past medical history presented to the Emergency Department with a 3-day history of high fevers, diarrhea, and malaise after hiking outdoors. He also visited a nearby hiking trail and swam in a local lake that contained “blue-green algae” and sewage waste. After he went home, his health continued to decline, and Emergency Medical Services was called due to his altered mentation and lethargy. He had no sick contacts, and he did not recall mosquito bites prior to his presentation. He had no significant past medical or surgical history. He did not take any medications and reported no allergies. There is no significant family history of autoimmune and neurological diseases. He denied alcohol, smoking, and recreational drug use. A review of systems showed no other pertinent findings. On admission, he was febrile to 103F, tachycardic to 150 bpm, tachypneic with a respiratory rate of 32 bpm, and hypertensive to 149/115 mmHg. BNP was 1,309 pg/ml. Troponin was elevated at 0.8 *μ*g/dl. Arterial blood gas showed a pH of 7.5 with pCO_2_ 29.7 mmHg and PO_2_ 54.3 mmHg, which demonstrated acute hypoxemia with respiratory alkalosis. Physical examination was notable for reduced lower extremity strength bilaterally with diminished deep tendon reflexes. He also had bilateral crackles and 2+ pitting edema at the lower extremities. He was immediately intubated due to rapidly declining oxygen saturation. For the next four days, his Glasgow Coma Scale score dropped from 15 to 5, with no additional sedation given following the 1st day of intubation. He continued to have progressive upper extremity weakness and hearing loss. Laboratory workup on admission showed a complete blood cell count with WBC of 22.6 k/mm^3^ with 91% neutrophils. Chest X-ray showed pulmonary edema with bilateral interstitial infiltrates (Figure 1). The complete metabolic panel was grossly unremarkable, except for hypokalemia at 3.0 mEq/dl. EKG demonstrated persistent sinus tachycardia without ST or T wave changes (Figure 2). No AV nodal blocks or other tachyarrhythmias were appreciated. A TTE showed left ventricular global hypokinesis with a reduced ejection fraction of 25-30% without valvular abnormalities (Figure 3). A lumbar puncture was performed, which showed pleocytosis with no evidence of bacterial infection, and the meningitis viral panel was negative (Cytomegalovirus, Enterovirus, Herpes Simplex Virus 1/2, Human Herpesvirus 6, Human Parechovirus, and Varicella Zoster Virus). Lyme, fungal, and Coxsackie viral serology were also negative. The initial WNV IgM using the antibody-capture enzyme-linked immunosorbent assay was negative (Manufacturer: ARUP, ID: 0050228, with clinical sensitivity of 98%, specificity of 92.4%).

**Figure 4:**
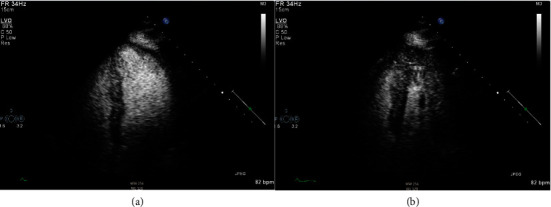
Left ventricular view of TTE with perflutren contrast demonstrates preserved LV ejection fraction with excellent contractility and the absence of thrombus prior to hospital discharge. (a) Left ventricle during diastole. (b) Left ventricle during systole.

At this time, the prevailing working diagnosis was understood to be Guillain-Barré Syndrome, and the patient was treated with IVIG and aggressive diuresis with furosemide. Due to a lack of improvement and worsening respiratory status on mechanical ventilation despite IVIG therapy, he was transitioned to plasmapheresis on hospital day 3. He was placed on norepinephrine and dobutamine due to the concern of mixed cardiogenic and septic shock in this young individual. When his flaccid paralysis and mentation progressively worsened despite plasmapheresis on day 5, we became increasingly concerned about WNV encephalitis. We retested for WNV serology, which ultimately came back IgM positive. Unfortunately, the virus neutralization test was unavailable at our institution. After 14 days of vasopressor/inotropic support, as well as five days of plasmapheresis, empiric antibiotics, and diuresis, the patient's mentation, pulmonary edema, and clinical status improved to the point where he was weaned off of mechanical ventilation. However, he continued to have significant upper and lower extremity weakness. Repeat TTE now showed EF > 50% with significantly improved global wall motion abnormalities (Figure 4). The patient was transferred to inpatient rehab shortly after extubation. No arrhythmias were detected throughout his hospitalization. He was followed up with his primary care physician, as well as a PM&R physician. Unfortunately, he continues to have residual lower extremity weakness 2 years after his initial hospitalization, and he is currently wheelchair-bound at the time of writing this article. Nevertheless, from a cardiac standpoint, he has complete recovery, and he is not on any medication for congestive heart failure.

## 3. Discussion

This case highlights a rare presentation of WNV meningoencephalitis complicated by acute viral myocarditis. WNV-associated myocarditis carries a poor prognosis because it can cause fatal arrhythmias, including asystole, ventricular tachycardia, and ventricular fibrillation due to inflammation and scar formation within the heart [4, 5]. However, cardiac involvement due to WNV infection is poorly understood. Based on the current literature, when the patient presents with both WNV meningoencephalitis and myocarditis, survival becomes increasingly unlikely, and mortality approaches near 100% [1–3, 5, 6]. Like most other viral myocarditis, there is no specific treatment for WNV myocarditis except for hemodynamic support, and the possible utility of IVIG and plasmapheresis. The most common causes of acute viral myocarditis are often adenovirus, enterovirus, parvovirus B19, and CMV, with a mortality ranging from 56 to 83 percent [7]. However, this is not the case for WNV myocarditis. In animals, there appears to be a preference for cardiac atrioventricular nodal tissues in WNV infection. In birds, the brain, heart, and lung often carry the highest viral titer. Hemolysin and eosin staining of bird cardiomyocytes infected with WNV also show significant necrosis and neutrophilic infiltration, indicating significant inflammation and scar formation that may lead to life-threatening arrhythmias [8, 9]. Prolonged sinus pauses have also been described in WNV hosts that eventually required temporary and permanent pacemaker placement [10]. Furthermore, animal studies also show that WNV targets the hyperpolarization-activated cyclic nucleotide-gated potassium channel 4 at the sinoatrial and atrioventricular node in mammals, predisposing them to autonomic dysfunction [11]. This may explain the high mortality rate observed in humans with WNV-associated cardiomyopathy.

The limitation of our case report is that we cannot distinguish our patient's new-onset congestive heart failure from stress-induced cardiomyopathy. In addition, we did not have viral deneutralization testing available at our institution. However, the lack of regional wall motion abnormalities involving the basal segment on TTE is not consistent with stress-induced cardiomyopathy. There were also no significant ST-T changes in the EKG on presentation. We also did not perform coronary angiography and/or left ventriculogram due to the lack of evidence and low pretest probability of coronary artery disease. The clinical presentation of tachycardia, new-onset CHF, and recent viral prodrome makes the diagnosis of WNV myocarditis very likely. The timing of recovery and resolution of left ventricular dysfunction is also more supportive of a viral inflammatory etiology (Figures 3 and 4). Cardiac MRI with late gadolinium enhancement may be helpful in determining the likelihood of life-threatening arrhythmias as it has been shown to have utility in the setting of acute viral myocarditis, as compared to more invasive measures such as endomyocardial biopsy [10].

Fortunately, our patient did not suffer from life-threatening tachyarrhythmias, as demonstrated in the current medical literature. However, patients with WNV myocarditis should remain on continuous cardiac telemetry and avoid clinical volume overload. Early IVIG and plasmapheresis may have helped in his recovery. Perhaps, a combination of unloading strategies such as mechanical circulatory support with ventricular assist devices, pulmonary artery catheter, and diuretic medication to reduce left ventricular end-diastolic pressure can be helpful to prevent tachyarrhythmias in the setting of cardiogenic shock [12]. Further cross-sectional and retrospective studies are required to understand the clinical outcome and prevalence of WNV myocarditis.

## Data Availability

All data is included in the case report.

## Additional Points

*Learning Objectives*. West Nile Viral myocarditis is a rare complication of WNV infection, and it carries a high mortality rate. Close cardiac monitoring and supportive care should be started promptly on presentation. Early steroid, intravenous immunoglobulin (IVIG), and plasmapheresis, as well as hemodynamic support, are imperative in the treatment of acute WNV myocarditis. Cardiac magnetic resonance imaging (MRI), ventricular assist device, and pulmonary artery catheter are useful tools to help guide diagnoses and treatment plans.
